# Lack of Rule-Adherence During Mountain Gorilla Tourism Encounters in Bwindi Impenetrable National Park, Uganda, Places Gorillas at Risk From Human Disease

**DOI:** 10.3389/fpubh.2020.00001

**Published:** 2020-02-13

**Authors:** Annalisa Weber, Gladys Kalema-Zikusoka, Nancy J. Stevens

**Affiliations:** ^1^Applied Research and Evaluation, Division of Global Health Protection, Center for Disease Control, Atlanta, GA, United States; ^2^Environmental Studies Program, Voinovich School for Leadership and Public Affairs, Ohio University, Athens, OH, United States; ^3^Conservation Through Public Health, Entebbe, Uganda; ^4^Department of Biomedical Sciences, Heritage College of Osteopathic Medicine, Ohio University, Athens, OH, United States

**Keywords:** gorilla, primate, tourism, ecotourism, disease transmission, Uganda, conservation

## Abstract

Mountain gorillas (*Gorilla beringei beringei*) are an endangered primate species, with ~43% of the 1,063 individuals that remain on the planet today residing in Bwindi Impenetrable National Park (BINP) in southwestern Uganda. These primates are at the heart of a growing tourism industry that has incentivized their continued protection, but close proximity between humans and gorillas during such encounters presents well-documented risks for disease transmission. The Uganda Wildlife Authority (UWA) has developed rules to help protect the health of the gorillas, limiting each habituated gorilla group to a single 60 min visit each day by a group of no more than 8 tourists, and emphasizing that humans maintain a >7 m distance from gorillas at all times. A number of studies have documented that not all tour groups respect these rules. This project assesses rule-adherence during gorilla tourism encounters at BINP using both observational and survey-based data collected during the tourism high season between May and August, 2014. Observational data from 53 treks reveal that groups of 1–11 tourists engaged in gorilla viewing encounters between 46 and 98 min in duration. Although 96% of pre-trek briefings conducted by park rangers emphasized the need to maintain >7 m human-gorilla spacing, the 7 m distance rule was violated in over 98% (52 out of 53) of the tours examined in this study. Observational data were collected at 2 min intervals during gorilla-viewing encounters, documenting the nearest distance between any tourist and a gorilla (*n* = 1,604), of which 1,094 observations (68.2%) took place at a distance less than or equal to 7 m. Importantly, the 7 m rule was violated in visits to all of the gorilla groups habituated during the time of the study. In 224 observations (~14%, per 1,604 total), human-gorilla spacing was 3 m or less. Survey data (*n* = 243) revealed promising opportunities to improve tourist understanding of and adherence to park rules, with 73.6% of respondents indicating that they would be willing to utilize a precautionary measure of wearing a face-mask during encounters to protect gorilla health.

## Introduction

Mountain gorillas (*Gorilla beringei beringei*) are an endangered primate species found in only two isolated forests of central-eastern Africa, spanning parts of Uganda, Rwanda, and the Democratic Republic of the Congo (1). Approximately 43% of the remaining mountain gorilla population inhabits Bwindi Impenetrable National Park (BINP)^1^ in southwestern Uganda, and since 1993, a robust tourism industry has developed around tourists viewing habituated gorilla groups.

The area around BINP is home to some of the densest human populations on the continent, for example, the Kisoro district is home to an average of 354 people per km^2^ (2). With one of the highest population growth rates in the world [population net gain of 1 person every 22 s, per (2)], Uganda faces long-term challenges for balancing the growing needs of the human population with desired outcomes in great ape conservation.

### History of Gorilla Tourism Around BINP

Gorilla-viewing tourism was introduced in 1993 as a potential solution to multiple problems, to generate revenue for the Ugandan government from gorilla viewing permits with a portion invested back into local communities, prioritizing gorilla conservation and offering continued protection of the Bwindi forest itself (3–6).

**Figure 1:**
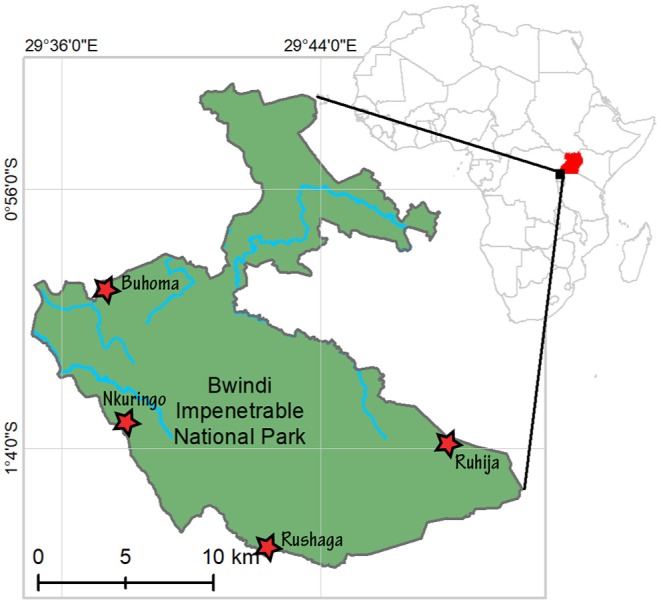
Study location: Bwindi Impenetrable National Park, Uganda. Stars indicate locations of trekking trail heads.

In the 1990's, approximately 3,000 people visited Bwindi annually. By 2011, BINP was receiving more than 15,000 tourists each year (7), and the numbers have continued to rise. Habituation of additional gorilla groups has been justified by increased tourist demand, and to spread the economic benefits of employment opportunities (e.g., through gorilla tourism jobs ranging from guides to porters to workers in the lodging/hospitality sectors) to more communities residing in different areas of BINP (Figure 1). The census conducted in 2018 estimated a minimum of 459 gorillas in Bwindi forming 50 family groups (1). In 2014, an estimated 42% of the BINP gorilla population was visited by the public, with habituation of 12 groups containing 168 gorillas. Indications from ongoing census efforts indicate that gorilla numbers in Bwindi remain stable, and the number of habituated groups had increased to 17 by the end of 2018 (1).

Habituating wild gorillas has been encouraged for both conservation and research purposes. The decision to habituate a gorilla group usually weighs costs and benefits (8–10). Benefits beyond revenue generation include protection of gorillas and their habitats, daily monitoring to identify any gorilla health issues, and facilitation of detailed research on behavior and ecology offering enhanced understanding of population dynamics including births, deaths, and dispersal patterns [e.g., (4, 11, 12)]. Costs of habituation include reduced avoidance of humans (13), potentially increasing the likelihood of crop raiding and/or susceptibility to poaching [e.g., (14–16)], and increased risk of disease spread by close proximity with humans and livestock (5, 7, 17–25). This risk is intensified by the high number of international visitors, generating potential for exposure to foreign infections to which local humans and great apes lack resistance (21, 26–31).

### Gorillas, Disease, and the 7 m Distance Rule

It has been well-documented that the profitable gorilla tourism industry promoted to facilitate continued protection of gorillas increases health risks by bringing thousands of people into close contact with endangered apes (7, 17, 18, 21, 32–39). Indeed, the close genetic relationship that gorillas share with humans renders them highly susceptible to human-borne illnesses, to which they have limited or no immunity (17, 21, 22, 35). For example, Graczyk et al. (34) reported on five juvenile gorillas from a habituated group in Bwindi, suffering from highly contagious scabies, similar to infections found in human populations surrounding the park. And in the early 2000's, western lowland gorilla populations decreased by a third after an outbreak of the Zaire strain of the Ebola virus (40), with additional concern raised by the potential for disease transmission between chimpanzees and gorillas (41). Increased contact between humans and wildlife intensifies the transmission of anthropozoonotic diseases and parasites (34), making tourists, along with high human population density around BINP habitats, a profound threat to the survival of mountain gorillas (5).

Infectious diseases (of which respiratory diseases are the most common) result in 20% of sudden deaths in mountain gorillas (20). Concern that apes are particularly susceptible to human respiratory infections has been well-documented [e.g., (22)], and studies have explored risks inherent in human-gorilla interactions [e.g., (5, 42, 43)]. Three respiratory-disease outbreaks among habituated chimpanzee populations within Cote D'Ivoire were traced back to two common human paramyxoviruses, through tissue sampling of deceased chimpanzees (19), and an outbreak of the respiratory disease human metapneumovirus (HMPV) was conclusively linked to the death of a female mountain gorilla in Virunga National Park that ultimately died of bacterial pneumonia, followed by the death of her infant likely due to neglect (20).

Diseases that spread from humans to gorillas without prolonged or direct physical contact are of grave concern, namely respiratory infections that are transmitted through the air (35, 38). Pneumonia, measles, tuberculosis, influenza, and RSV (respiratory syncytial virus) infections are considered particularly dangerous (21). Habituated gorilla groups face potential exposure to nearly 50,000 visitor-hours per year at Bwindi should all 17 currently-habituated groups eventually be trekked at full capacity of 8 one-hour visitors per day, not to mention exposure to trackers, guides, guards, doctors, and rangers. Put another way, tourism exposes habituated gorillas to more humans per year than would visit an average person's house throughout an entire lifetime (44).

In order to mitigate health risks to both gorilla and human populations, the Uganda Wildlife Authority has developed rules to help protect the health of the gorillas, limiting each habituated gorilla group to a single 60 min visit per day by a group of no more than 8 tourists (with the insistence that sick individuals do not trek), and emphasizing that humans maintain a >7 m distance from gorillas at all times. Of concern is how consistently these rules are applied (5, 37, 42, 45, 46). For example, Adams et al. (47) reported that although 69% of tourists visiting chimpanzee populations were aware that ill individuals should abstain from chimpanzee treks, 17.7% of people engaging in the treks had symptoms of active infections. And a full 25% of tourists surveyed during a study in 2011 admitted willingness to trek gorillas at Bwindi while ill, noting reluctance to give up an expensive experience and challenges with rescheduling (5). The same study documented that only about 51% of tourists at that time reported they were willing to don protective face-masks to protect gorilla populations from human-transmitted diseases. Sanbrook and Semple (42) reported that gorillas and humans interacted in closer proximity than the stipulated 7 m, for example as close as 2.8 m. A study conducted in 2011 reported instances of direct contact between humans and gorillas, including those who reported being ill (5). Not all guides are equally versed in the rationale behind the rules for safely leading tourists groups, which can result in irregular enforcement of park regulations (48).

The rationale for the minimum-distance rule that human-gorilla proximity should never be closer than 7 m relates to the particular susceptibility of gorillas to airborne infections, and the fact that a sneezed droplet containing infectious particles can cross a distance of 6 m in a controlled indoor environment (49, 50). The degree of potential disease or infection exposure is related to the probability of contact between an infected human and a gorilla, exposure duration, and the infectivity factor of the source, a function of the germ carrier's status, the stage of the infection, and the source's behavior, such as uncovered coughs and sneezes (44). Even light wind can substantially boost travel distance for aerosol particles, influencing exposure risk for gorillas dramatically if the wind is blowing in the direction of the gorillas (44).

### Are Masks a Potential Solution?

One way to reduce the amount of disease-carrying aerosol particles released into the atmosphere is to introduce a rule requiring tourists to wear protective face-masks during the tourism experience. Indeed, wearing protective masks is considered best practice among scientists working in primate conservation (19, 21, 51) and this measure is already in place in The Democratic Republic of the Congo, where tourists regularly wear protective face-masks during gorilla tourism encounters. Although only about half of tourists surveyed in 2011 reported that they would be willing to don a protective facemask to protect gorillas (5), additional data are required to revisit whether mask-wearing presents a viable solution for mitigating disease transmission in BINP at this time.

### Objectives of This Study

Existing research on gorilla tourism suggests that additional data are also needed in order to better assess whether irregularities in rule adherence occur that could intensify risk of human-gorilla disease transmission risk during tourism encounters. As previously noted, BINP rules stipulate that only 8 tourists may trek a particular gorilla group each day, with tourism encounters limited to 60 min, and human-gorilla spacing >7 m. Based on previous studies and our own observations, we hypothesize tourists will break the 7 m rule by coming in closer contact with gorillas during their trek, regardless of being briefed beforehand to maintain this distance. In 2014, our team set out to explore 7 m rule adherence in gorilla tourism encounters at Bwindi, recording also the number of tourists per trekking group and the duration of the gorilla viewing encounter. Possible explanations for 7 m rule violations include that tourists may be unaware of the rule, they may violate the 7 m rule accidentally, they may choose to/be encouraged to move closer than 7 m to better view the gorillas, and/or habituated gorillas may themselves approach tourists. This study documents adherence to Uganda Wildlife Authority gorilla-trekking rules and regulations during tourism encounters in BINP through the evaluation of field-based observations of informational briefings and subsequent tourist—gorilla spacing, together with a questionnaire survey administered to explore tourist perception and attitudes about disease transmission and prevention measures.

## Study Site and Methods

### Study Site

Bwindi Impenetrable National Park (BINP) is located in southwest Uganda in eastern Africa (Figure 1). The area has received focused governmental protection since 1932, and by 1964, the reserve was given added protection as an animal sanctuary in order to protect the mountain gorillas living within the area. It was officially gazetted as a national park in 1991, with regulated gorilla trekking commencing in April 1993.

### Data Collection

Data collection for this study was conducted in BINP by accompanying tourist groups during their pre-trek briefings and gorilla viewing visits, and included the following observations: number of tourists per group, number of individuals per trek (including tourists, guides, guards, porters, researchers), content of pre-trek briefings by BINP staff (specifically, whether or not the 7 m distance rule was explicitly noted by park staff, and whether human–gorilla disease transmission risks were discussed), duration of “gorilla hour” viewing encounter (according to the rules of BINP, once tour groups have trekked to the location of a habituated gorilla group, the amount of time tourists are allowed to spend viewing gorillas is limited to 60 min), duration of trek (which varied depending on starting point, local topography, and distance from gorilla group; trek here is defined as the travel from park headquarters to the gorilla viewing area, plus the “gorilla hour,” plus the travel back to the park headquarters), and estimated distances between gorillas and humans during the viewing encounters (including tourists and park staff). Occasionally the number of non-tourists (tourists, guides, guards, porters, and researchers) accompanying the group fluctuated during an experience; these cases were recorded as missing data. Data were collected by AW during 53 separate gorilla trekking tourism encounters, sampling visits to all 12 gorilla groups habituated at the time of the study, with densest sampling focused on the three most-frequented Buhoma groups: Habinyanja group (*n* = 15 treks), Mubare group (*n* = 14 treks), and Rushegura group (*n* = 12 treks).

During all gorilla tourism encounters, scan estimates of human-gorilla proximity were conducted at 2 min intervals recording the smallest distance between any one human and any one gorilla in the tourism encounter. Human-gorilla distances recorded during 2-min scans were placed into 11 distance categories: <1, 1.1–2, 2.1–3, 3.1–4, 4.1–5, 5.1–6, 6.1–7, 7.1–8, 8.1–9, 9.1–10, and >10 m, as human–gorilla spacing could not be accurately estimated at a distance over 10 m due to dense vegetation in the study area. Prior to the study, the estimation of vertical, horizontal, and oblique distances were practiced and independently validated in habitats of differing complexities using calibrated measurement distances to ensure validity and reliability of distance estimates in the variable habitats of the gorilla groups visited by the tourist groups. It was occasionally necessary to shift position in order to record the distances between humans and gorillas through the obscuring vegetation. Occasionally it was not possible to record an observation due to movement patterns of the tourists/tourist group and/or obscuring vegetation; those cases were recorded as missing data. At no time did we violate the 7 m rule ourselves in collecting the data for this study.

### Assessing Adherence to the “7 m Distance” Rule

Data on human-gorilla spacing were organized based on the date of the trek and the name of the gorilla group visited. The number of 2-min scan observations of human-gorilla spacing were summed by distance categories and divided by the total number of observations in that trek to obtain proportional data for each distance category. The time at first 7 m distance violation from the beginning of each gorilla encounter was recorded. The number of sequential distance violations in a given gorilla-viewing encounter was calculated as a proxy for duration of violation. When the distance between humans and gorillas dropped to 3 m or less, it was recorded, whether or not it coincided with 2 min scan observations. It was also noted whether it was a human that closed the distance from a gorilla, or vice-versa, for comparison with such close interactions as recorded by Sanbrook and Semple (42).

Because we hypothesized a greater opportunity for rule violations in groups with more tourists, and that tourists trekking for longer to view a given gorilla group might feel more entitled to move closer to the gorillas, Spearman rank correlations were calculated to assess relationships between the proportion of 7 m rule violations out of the total observations in a given trek and the following variables: number of tourists per trekking group, and duration of the trek (significance levels set at *p* = 0.05). Linear logistic regression models using a quasi-binomial family to account for over-dispersion of proportional data were run through the statistical analysis platform R to examine the relationship between the response variable (proportion of rule violations out of the total observations in a given trek) and the following predictor variables: number of tourists in trekking group, and duration of the trek.

### Evaluating the Knowledge, Perceptions and Attitudes of Tourists Toward the Rules of BINP

For each trek, we documented content of the mandatory pre-trek briefing provided by the staff of BINP, namely: (1) whether the 7 m distance rule was mentioned in the briefing (yes or no), and (2) whether human–gorilla disease transmission risks were discussed (yes or no).

Subsequent to the gorilla-viewing encounters, tourists were asked to complete a voluntary five question survey. The questionnaire gathered yes/no data on tourist knowledge and attitudes regarding ecotourism rules relating to health and safety, including (1) whether tourists believed they were educated about the 7 m rule, (2) whether they believed that they maintained a 7 m distance from the gorillas, (3) whether they thought that such a rule was necessary, (4) whether the rule detracted from their tourism experience, and (5) whether or not they would be willing to wear a protective face-mask during gorilla viewing to prevent transmission of infections. A smaller number of tourists responded to the fifth and final question as it was added to the survey partway into the study at the request of coauthor GKZ; every participant who received this question on the survey provided a yes/no response. The survey was administered in English, by far the most widely spoken language among tourists in the study, hence a notable limitation of the present study is that it only examines responses of English-speaking tourists. Only adults 18 years and older were asked to complete survey questionnaires.

Aggregate data on self-reported knowledge and attitudes were gathered from tourist survey responses and discussed in the context of observed nearest human-gorilla distances during the study, as well as with percentages of pre-trek “rules and regulations” briefings delivered by BINP staff that addressed the 7 m rule and risks of human-gorilla disease transmission, in order to explore the need for improved communication about safe tourist-gorilla spacing during gorilla-viewing treks.

## Results

### Tourist Informational Briefings by Park Staff

**Figure 2:**
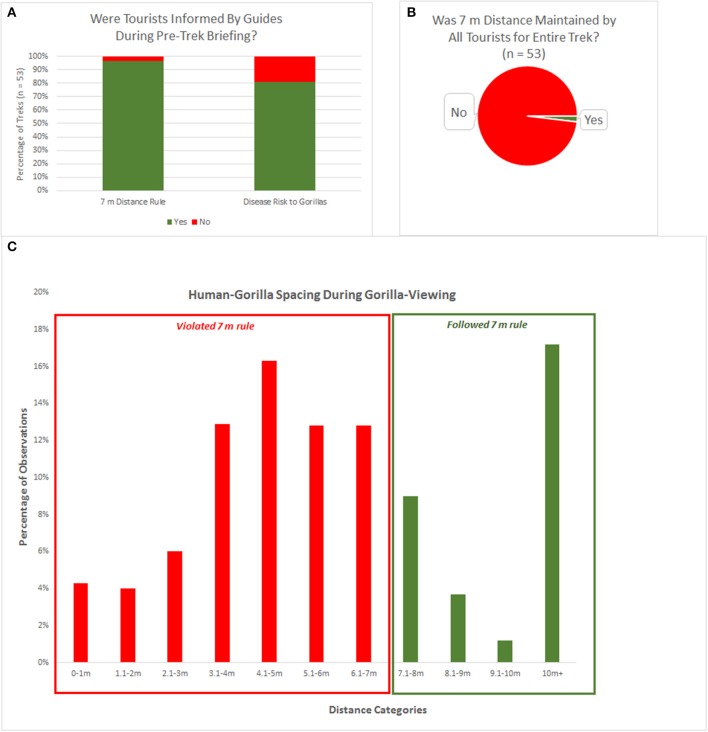
Rule adherence information and behavior during gorilla viewing. **(A)** Percentage of treks in which tourists were informed by guides of the 7 m rule and the importance of maintaining that distance to protect gorillas from disease transmission. **(B)** Percentage of treks with rule adherence vs. violations (*n* = 53 treks). **(C)** Observations of nearest human-gorilla spacing collected at 2 min intervals, shown by distance category.

Gorilla trek observations were conducted 53 times between 25 May and 8 August 2014. Pre-trek briefings were delivered in English and varied notably in length and content, generally providing information on the history of the park, the history of gorilla habituation, rules that tourists are expected to follow during the trekking experience, and the reasons why the rules are in place. The overwhelming majority of pre-trek briefings examined in this study, 96.2% (51/53) indicated the rule of maintaining a distance of at least 7 m from the gorillas at all times, and 81.1% (43/53) discussed the risk of disease transmission between humans and gorillas in close proximity (Table 1 and Figure 2A).

**Table 1:** Data collected during gorilla tourism encounters in BINP.

**Date**	**Group**	**7 m rule brief**	**Disease-risk brief**	**Trek duration**	**Visit duration**	**#Tourists**	**Total # people**	**Under 7 m?**	**Under 3 m?**
May 25, 2015	Oruzogo	No	No	173	60	7	13	Yes	No
May 26, 2015	Oruzogo	Yes	Yes	150	66	7	13	Yes	No
May 27, 2015	Kyagurino	Yes	No	185	66	5	11	Yes	Yes
May 28, 2015	Oruzogo	Yes	Yes	148	65	7	13	Yes	No
May 30, 2015	Bitukura	Yes	No	220	67	8	13	Yes	Yes
May 31, 2015	Kyagurino	Yes	No	258	58	4	11	Yes	Yes
June 2, 2015	Habinyanja	Yes	No	307	81	8	13	Yes	Yes
June 3, 2015	Mubare	Yes	Yes	154	83	6	12	Yes	Yes
June 4, 2015	Habinyanja	Yes	No	259	76	8	18	Yes	Yes
June 5, 2015	Rushegura	Yes	No	246	84	6	21	Yes	Yes
June 7, 2015	Habinyanja	Yes	Yes	167	68	8	NA	Yes	Yes
June 8, 2015	Mubare	Yes	Yes	182	69	8	19	Yes	Yes
June 12, 2015	Bweza	Yes	Yes	209	58	7	16	Yes	Yes
June 13, 2015	Mishaya	Yes	Yes	375	70	8	17	Yes	Yes
June 14, 2015	Nkuringo	Yes	Yes	235	65	4	9	Yes	No
June 16, 2015	Rushegura	Yes	Yes	215	64	1	6	Yes	Yes
June 17, 2015	Habinyanja	Yes	Yes	244	78	7	19	Yes	Yes
June 18, 2015	Mubare	No	No	206	80	4	13	Yes	Yes
June 19, 2015	Mubare	Yes	Yes	267	67	7	13	Yes	Yes
June 20, 2015	Mubare	Yes	Yes	181	46	8	18	Yes	Yes
June 21, 2015	Habinyanja	Yes	Yes	197	61	8	18	Yes	Yes
June 22, 2015	Mubare	Yes	Yes	210	60	8	19	Yes	Yes
June 24, 2015	Habinyanja	Yes	Yes	202	98	8	18	Yes	Yes
June 25, 2015	Habinyanja	Yes	Yes	249	65	6	11	No	No
June 26, 2015	Mubare	Yes	Yes	178	62	11	23	Yes	Yes
June 27, 2015	Rushegura	Yes	Yes	285	69	8	18	Yes	Yes
June 28, 2015	Mubare	Yes	Yes	212	71	8	20	Yes	Yes
June 29, 2015	Rushegura	Yes	No	293	56	5	15	Yes	Yes
July 1, 2015	Nshongi	Yes	Yes	325	73	2	7	Yes	No
July 2, 2015	Kahungye	Yes	Yes	338	68	8	13	Yes	No
July 3, 2015	Busingye	Yes	Yes	325	71	3	7	Yes	No
July 4, 2015	Habinyanja	Yes	Yes	89	54	7	NA	Yes	Yes
July 5, 2015	Habinyanja	Yes	Yes	220	58	8	NA	Yes	Yes
July 6, 2015	Mubare	Yes	Yes	213	79	8	20	Yes	Yes
July 8, 2015	Rushegura	Yes	Yes	349	65	7	17	Yes	Yes
July 9, 2015	Mubare	Yes	Yes	158	67	10	17	Yes	Yes
July 22, 2015	Rushegura	Yes	Yes	101	54	8	21	Yes	Yes
July 23, 2015	Rushegura	Yes	Yes	231	64	8	22	Yes	Yes
July 24, 2015	Habinyanja	Yes	Yes	338	66	8	18	Yes	Yes
July 25, 2015	Habinyanja	Yes	Yes	97	71	8	17	Yes	Yes
July 26, 2015	Mubare	Yes	Yes	267	69	8	17	Yes	Yes
July 27, 2015	Habinyanja	Yes	Yes	168	66	8	18	Yes	No
July 28, 2015	Rushegura	Yes	Yes	411	66	8	21	Yes	Yes
July 29, 2015	Mubare	Yes	Yes	219	60	6	NA	Yes	Yes
July 30, 2015	Rushegura	Yes	Yes	282	61	8	18	Yes	Yes
August 1, 2015	Mubare	Yes	Yes	286	87	10	21	Yes	Yes
August 2, 2015	Habinyanja	Yes	Yes	130	76	8	21	Yes	Yes
August 3, 2015	Rushegura	Yes	No	231	54	8	19	Yes	Yes
August 4, 2015	Rushegura	Yes	Yes	257	66	8	18	Yes	Yes
August 5, 2015	Mubare	Yes	Yes	204	71	8	20	Yes	Yes
August 6, 2015	Habinyanja	Yes	Yes	279	64	8	19	Yes	Yes
August 7, 2015	Rushegura	Yes	Yes	105	54	8	19	Yes	Yes
August 8, 2015	Habinyanja	Yes	Yes	295	61	8	18	Yes	Yes

### Evaluating Adherence to the “7 m Distance” Rule and Instances of Close Proximity

Of the 53 tourism encounters examined in this study, only a single trek lacked violations of the 7 m distance rule by any tourist for the entirety of the gorilla viewing experience (Table 1 and Figure 2B). Of the 1,604 nearest-neighbor observations, 1,094 observations took place at a distance less than or equal to 7 m, i.e., 31.8% of observations were in accordance with the 7 m distance rule, and 68.2% of observations violated the 7 m distance rule (Figure 2C). In all observations in the study, tourists were the closest humans to the gorillas, although guides were often very near the closest tourist, moving vegetation away from the line of sight or pointing to the nearest gorilla.

Notably, 277 all-occurrence observations recorded human–gorilla spacing at a distance of 3 m or less, 224 of these observations occurring at 2 min sampling intervals. Of these violations, 165 instances (59.6%) were initiated by gorillas, and 112 instances (40.4%) were initiated by tourists; of the latter, some instances were facilitated by guides, in order to obtain a clear line of sight for photography. Human–gorilla spacing at <3 m was recorded during tourism encounters with eight of the 12 gorilla groups habituated at the time of the study, including five groups trekked only once or twice during the study (Table 1). Human–gorilla spacing at <3 m was *not* observed during treks with the following groups: Oruzogo (3 treks), Nkuringu (1 trek), Nshonge (1 trek), and Busingye (1 trek), although the 7 m rule *was* violated in all of the treks with those four groups (Table 1).

### Number of Tourists per Group, Duration of Trek, and Duration of “Gorilla–Hour” Viewing Experience

The number of tourists per gorilla encounter averaged 7 (sd = 2, range = 1 tourist−11 tourists), with the total number of people per trek including park staff averaging just over 16 (sd = 4.15, range = 7–23 people). Overall trek durations (trekking to and from gorilla viewing position plus gorilla viewing time) were highly variable across tourist groups, averaging 229 min (sd = 72.07, range = 89–411 min). In this study, the actual amount of time tourists spent viewing gorillas also varied. The average “gorilla hour” was 66 min in duration (sd = 9.44, range = 46–98 min).

Spearman rank correlation analysis revealed a weakly positive but non-significant relationship (r_s_ = 0.15; *p* = 0.269) between the proportion of rule violations in a given trek and the number of tourists within that trekking group, and a marginally significant negative relationship between the proportion of rule violations in a given trek and duration of the time spent trekking to the gorilla-viewing location (r_s_ = −0.27; *p* = 0.052). No significant predictors of rule violations were recovered by logistic regressions performed in R predicting the response variable (proportion of rule violations out of the total observations in a given trek), based on number of tourists per trekking group and duration of the trek to the gorilla-viewing location (Supplementary Table).

### Timing and Duration of 7 m Rule Violation During Tourism Encounters

**Figure 3:**
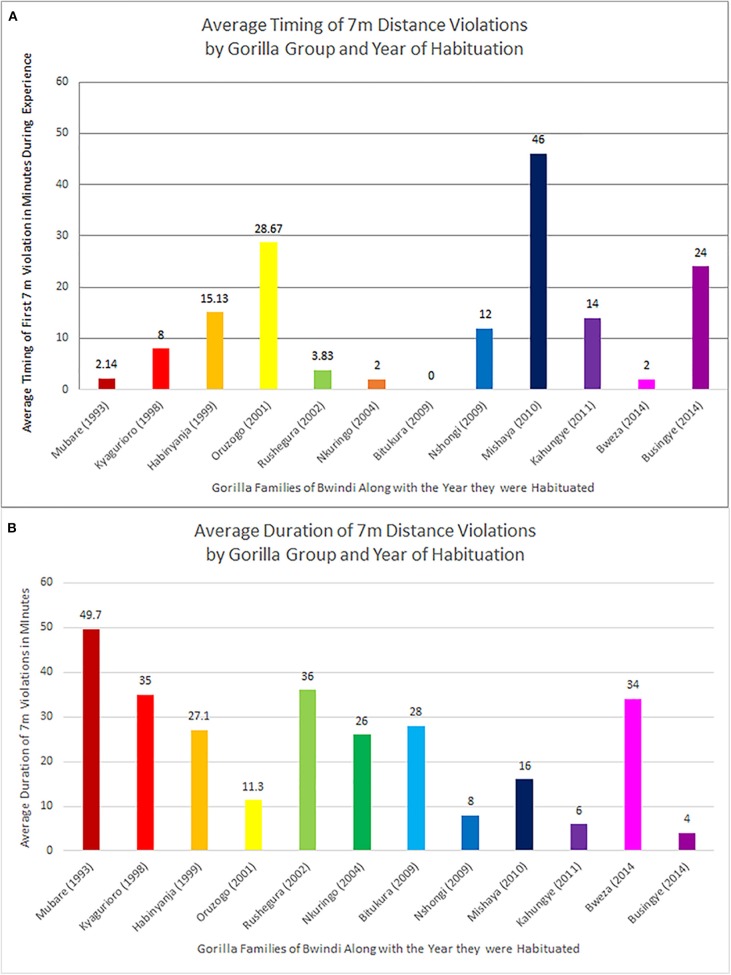
Timing and duration of 7 m rule violations for human-gorilla spacing during this study, by gorilla group and year of habitation. **(A)** Average time in minutes until first 7 m rule violation during an hour-long viewing experience, as a function of gorilla group. **(B)** Average duration in minutes of 7 m rule violations during an hour-long viewing experience, as a function of gorilla group.

During the 52 encounters with 7 m rule violations, the first violation of the 7 m distance rule occurred on average 9.8 min into the encounter (sd = 13.8 min; range = 0–46 min), with considerable variation in the time until and duration of rule violations in the different gorilla groups observed (Figure 3A). The three groups trekked most frequently in this study were Habinyanja group (*n* = 15 treks), Mubare group (*n* = 14 treks), and Rushegura group (*n* = 12 treks). Of these, encounters with the Mubare group (habituated in 1993) tended to have the highest percentage of nearest-neighbor observations with human-gorilla spacing that violated the 7 m rule, at over 85% of the total observations across treks. Next highest was Rushegura group (habituated in 2002) with 76% of observations comprising 7 m distance violations, followed by Habinyanja group (habituated in 1999), with over 58% of the total number of observations of the group comprising 7 m distance violations (Table 1).

Once the 7 m distance between humans and gorillas was breached, the duration of the violation varied from 2 to 70 min (some also violating the gorilla viewing duration of 60 min). The 70-min violation occurred during an encounter with the Mubare group, during which all 2 min sampling observations recorded human-gorilla spacing at a distance <7 m. On average, the duration of time when tourists were positioned <7 m to the gorillas during the “gorilla hour” viewing experiences sampled in this study was 43.5 min (Figure 3B).

### Tourist Perceptions and Attitudes About Gorilla Tourism Encounters

**Figure 4:**
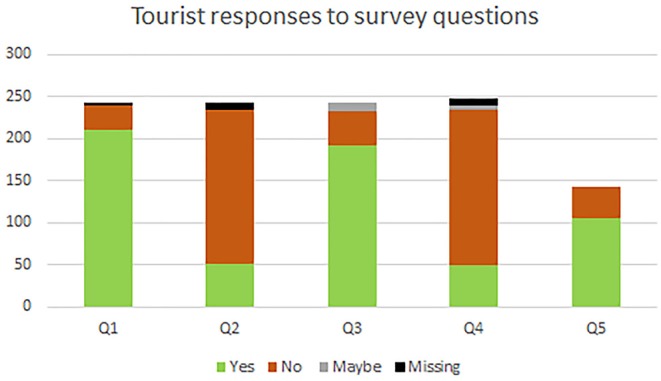
Tourist responses to survey items on study questionnaire. (Q1) Did you know about the rule limiting human-gorilla proximity to 7 m? (Q2) Do you think a 7 m distance was maintained between yourself and the gorillas? (Q3) Do you think it is necessary for people to maintain a distance of 7 m from the gorillas? (Q4) Do you think the 7 m distance rule detracts from the gorilla-viewing experience? (Q5) Would you be willing to wear a face-mask during the gorilla-viewing experience? (in these charts, green indicates “yes,” orange indicates “no,” gray indicates “maybe/uncertain,” and black indicates no answer).

Of the 379 tourists accompanied during the study, approximately 64% elected to complete post-trek surveys, yielding a total of 243 surveys included in the study. Not all survey respondents answered every question; results are depicted in Table 2 and Figure 4. Of the 239 respondents to the first question, 211 responded that they were aware of the 7 m distance rule, and 28 reported that they were not. Of the 235 respondents to the second question, 52 responded that they believed they kept a 7 m distance from gorillas, whereas 183 responded that they had not. Of the 243 respondents to the third question, 192 reported that they believed that it is necessary to maintain a 7 m distance from the gorillas, 41 reported that they did not believe it was necessary, and 10 responded that they were unsure. Of the 235 respondents to the fourth question, 50 thought that the 7 m rule detracts from the visitor experience, 185 did not feel that maintaining a 7 m distance from gorillas would detract from the experience, and 5 respondents were unsure. Because the fifth question was added in partway through the study, only 142 people received it in their survey and all responded; 106 reported that they would be willing to wear a face-mask to protect the health of the gorillas whereas only 36 reported that they would be unwilling to do so.

**Table 2:** Survey questionnaire questions and responses.

**Question**	***N***	**Yes**	**No**	**Maybe**	**Missing**	**% Yes**	**% No**	**% Maybe**	**% Missing**
(1) Did you know about the rule limiting human–gorilla proximity to 7 m?	239	211	28	0	4	88.28	11.72	0	1.67
(2) Do you think a 7 m distance was maintained between yourself a n d the gorillas?	235	52	183	0	8	22.13	77.87	0	3.40
(3) Do you think it is necessary for people to ma intain a dista nce of 7 m from the gorillas? 233	233	192	41	10	10	82.40	17.60	4.29	4.29
(4) Do you think the 7 m distance rule detract s from the gorilla-viewing experience?	235	50	185	5	8	21.28	78.72	2.13	3.40
(5) Would you be willing to wear a face-mask during the gorilla-viewing experience?	142	106	36	0	0	74.65	25.35	0	0

## Discussion

### Improving Tourist Knowledge, Perceptions, and Attitudes About Behavior During Gorilla Encounters

Our study hypothesized that the 7 m distance rule would not be uniformly and consistently followed during tourism encounters at BINP. Based on data from 53 gorilla viewing tourism treks incorporating all habituated gorilla groups at the time of the study, our hypothesis was supported. The 7 m distance rule was only upheld in 2% (1/53) of treks in this study, whereas in 98% of treks tourists came into closer contact than 7 m, breaking the rule, in 67 observations (~4% of 1,604 observations) within a meter of gorillas.

For gorilla encounters, the pre-trek briefing at park headquarters is important, because it is the only formal opportunity for an explicit discussion regarding rules and regulations of BINP available to shape tourist knowledge and attitudes about the experience. Although 51 out of 53 pre-trek briefings (96.2%) included information regarding the importance of maintaining a distance of 7 m from the gorillas, over 11% of tourists reported after the trek that they had been unaware of the 7 m rule (Table 2). Several potential reasons could explain why an individual tourist could embark upon a trek without knowing about the 7 m distance rule and its critical role in protecting the health of gorillas. It is possible that some tourists may have missed all or part of the pre-trek briefing. Even if they were present at a pre-trek briefing where this information was provided, tourists may not retain information from the briefing due to distraction; the morning briefing takes place at park headquarters, where it is often noisy and crowded, and filled with many people and vehicles. Moreover, the briefing takes place outside in a somewhat dispersed setting. There is the potential for a language barrier to impact understanding of rules related to human-gorilla spacing, and this could be explored in future studies. Although the all-Ugandan BINP staff are fluent in English, most tourists were international and some speak English as a second language or not at all. There is also the simple possibility that tourists are not attentive during the briefing, or that they are not accurately self-reporting their knowledge.

When tourists were asked whether they perceived that it is necessary to maintain a 7 m distance from gorillas, 16.9% of respondents answered “no” and an additional 4.12% of respondents indicated that they were unsure. This finding may reflect that not all morning briefings in this study mentioned the risk of disease transmission between humans and gorillas that can occur in close proximity between the two species. Disease transmission was mentioned in 43 out of 53 (81%) tourist briefings. At times, tourists were instructed to maintain a 7 m distance from the gorillas in order to reduce gorilla “stress and disturbance.” A potential problem with this approach is that tourists cannot be expected to adequately assess stress in habituated gorillas, making it a less effective a deterrent than simply emphasizing risks of disease transmission.

Nonetheless, differences are apparent between information presented in morning briefings and self-reported tourist knowledge, attitudes, and perceptions. These differences could be addressed through more deliberate and consistent messaging from BINP staff at morning briefings, by updating the gorilla tracking video shown to tourists before the morning briefing, and by providing written handouts that explain risks and visitor rules. Developing a set script for staff members could formalize the morning briefing and help ensure that the “7 m distance” rule and that risks of disease transmission between humans and gorillas are clear to all tourists. Indeed this has been suggested in previous studies [e.g., (5, 51)], so it would be interesting to explore why a set script has not yet been formally adopted. In addition, we advocate for a second briefing to be instituted immediately before the tourists begin gorilla viewing (i.e., when they have located gorillas but before approaching them) in order to emphasize that the 7 m distance must be maintained between humans and gorillas at all times, and that this rule is in place to help mitigate potential disease risk to the endangered gorillas. Increasing the efficacy of communication between tourists and park staff of BINP represents an essential yet simple tool for increasing rule adherence and safe ecotourism practices.

Importantly, even though 88.3% of tourist respondents indicated that they were aware of the 7 m distance rule, and 79.01% indicated they thought it was important to gorilla health to maintain that 7 m distance, only 22.1% reported having maintained a 7 m distance during their own gorilla viewing experience, indicating a significant disconnect to be addressed regarding rule adherence and rule enforcement during gorilla encounters during this study. Rule disregard and lack of enforcement have been well-documented in gorilla tourism studies for decades [e.g., (42)]. We recommend that tourist briefings be clarified and repeated to emphasize the health risks posed to gorillas by proximity to humans at a distance <7 m, and that park staff be encouraged and empowered to consistently and effectively enforce the 7 m rule.

### In Reality: Tourists Are Too Close to Gorillas

Tourist perceptions aside, this study shows that although 96% of tourists were informed of the 7 m rule preceding their trek, tourists broke this rule in 98% (52/53) of the treks assessed. This pattern was consistent across headquarter locations in the park, throughout the tourist season, and across all gorilla groups. Rule violations occurred in small and large groups of tourists (some group sizes also exceeding the rule of 8 tourists per trek), and in treks of the shortest and longest durations (with a majority of treks lasting longer than the 60 min stipulated for gorilla viewing experiences). Notably, 7 m rule violations often began early during gorilla viewing and persisted without correction; 7 m violations occasionally persisted for the entire viewing period. These violations suggest BINP staff and guides were reluctant to intervene to correct the problems and imply that some gorillas may be accustomed to close human proximity such that they do not move away from tourists even when in proximity close enough for disease transmission (indeed, gorillas approached humans in over half of the <3 m encounters). It would be interesting for future studies to explore differences in rule violations based on the time since habituation and composition (including number of juveniles) of gorilla groups. Environmental factors during treks probably also influence human-gorilla proximity (e.g., steep terrain, narrow pathways, and key vantage points), as they can at times make it difficult or impossible for humans to move away from gorillas.

In 81% of morning briefings, tourists were informed of the health risks of violating the 7 m distance rule, and in most instances, tourists were briefed that if the distance was closed by gorillas, it was the tourists' responsibility to move away to maintain the minimum 7 m distance. If humans are approached by gorillas and appear reluctant to increase the distance between themselves and the animals, then park staff must have strategies to successfully intervene. Yet park staff are placed in a potentially difficult position when it comes to enforcing the rules of BINP. Gorilla trekking is an expensive, rare opportunity, and tourists who engage in these treks often travel a great distance to do so, thus they may have expectations and feelings of entitlement that are unrealistic for a wild animal viewing experience (51). Hence, Bwindi guides are placed under intense pressure to provide tourists with an exceptional time-limited viewing experience of an endangered primate species. Of course, the livelihoods of Bwindi guides can also be enhanced by tips from tourists, adding to the pressure of ensuring tourist satisfaction with the experience, and perhaps generating reluctance about negatively impacting tourist satisfaction. It would be interesting to explore variation in guide responses to 7 m rule violations. Some guides may strive to reinforce the 7 m distance rule, whereas other guides may be hesitant to interfere with or upset the tourists. The complexities of economic disparities and inequities in the wildlife tourism industry have been deeply explored elsewhere [e.g., (52–61)], but the bottom line is that as gorilla tourism continues to expand, enforcement of the 7 m rule remains problematic in long-habituated as well as newly habituated gorilla groups at BINP.

### Mask the Problem?

The tourism industry at BINP continues to present a conservation paradox, with steady revenue generated through tourists visiting gorillas a powerful incentive to continue protecting the habitat and incredible biodiversity found within the park. But the risk of disease transmission between humans and gorillas poses a real threat to continued mountain gorilla survival, and this study demonstrates that tourists are still generally not adhering to the “7 m distance” rule in place to protect mountain gorilla health. Gorilla conservation is reliant upon tourism (11), yet the current gorilla tourism model is still not ensuring safe human-gorilla interactions.

One potential solution to mitigate the present risks is to introduce a rule requiring tourists to wear protective face-masks during the gorilla encounter experience to reduce the amount of disease-carrying aerosol particles released into the atmosphere (62). In the final survey question “Would you be willing to wear a face-mask during the gorilla-viewing experience?” the majority of respondents (73.6%), indicated they would be comfortable with the introduction of this practice. This figure is important, and higher than the 51% positive responses from tourists reported in a 2011 survey (5). Wearing protective masks is considered best practice among conservation scientists (19, 21, 51) and is currently in place in The Democratic Republic of the Congo, where tourists regularly wear protective face-masks during gorilla tourism encounters. Based on best practice recommendations for mitigating transmission of respiratory diseases between humans and great apes (21, 51), we recommend here that tourists be required to use face-masks (e.g., N95 surgical respirator masks) during gorilla encounters at BINP to reduce the risk of disease transmission.

### Long-Term Strategy: Empowering Guides and Managing Tourist Expectations

To increase tourist knowledge and promote responsible tourism practices, sustainable long-term strategies must urgently aim to improve human–gorilla spacing, empowering guides through staff training exercises that target and practice strategies for intervention that maintain a positive tourist experience without compromising gorilla health. At the time of this study, *all habituated gorilla groups in BINP were exposed to tourists at a distance* <*7 m*. Shaping tourist expectations and guide responses to rule violations may benefit from focused mediation training for staff, including managing tourists from different cultures. This can help guides to address challenges and better direct human behavior during gorilla trekking experiences. Providing additional resources and training to BINP staff can help improve confidence, empowering staff to better enforce the “7 m distance,” rule.

Importantly, Macfie and Williamson (51) point out the need for managing tourist expectations at the outset of a viewing experience. One approach may be to adjust tourist expectations about the gorilla tourism experience altogether. For example, successful conservation of wildlife habitat has been facilitated by wilderness-style experiences along the Kinabatangan River in Malaysian Borneo that feature simply the “possibility” of viewing orangutans (51) (NJS pers. obs.). This raises the potential for a deliberate gradual shift of the tourism experience made available by the Uganda Wildlife Authority to highlight more sustainable and meaningful opportunities for tourists to “search for” gorillas (for example at a distance far >7 m), rather than marketing more or less guaranteed photo opportunities at close range. Notably, orangutan tour operators rarely guarantee orangutan sightings, but tourists still visit. Communicating similar expectations to tourists interested in viewing gorillas could help shift distances back over time, and in conjunction with such a shift, communications could emphasize other fauna and flora that may be seen during treks. Long-term shifts to a less intensive gorilla viewing experience could not only serve as a sustainable solution to mitigate diseases spread by close encounters, it may also reduce gorilla over-habituation, and hence human-gorilla conflict, as less habituated animals may be less inclined to venture into areas of human occupation for crop raiding (51). Long-term strategies can prioritize a more sustainable experience, where the goal would be “to catch an exciting glimpse” of the gorillas rather than to spend an hour in extremely close proximity to them, taking similar pictures over and over.

Gorilla tourism at BINP has proven successful in protecting forested habitat and conserving endangered mountain gorilla populations over the past two decades, at the same time providing livelihoods and development opportunities for local communities. As human populations continue to grow and gorillas become increasingly habituated, however, communities must adopt new and more effective strategies if they wish to conserve endangered great ape populations and sustain gorilla-viewing tourism into the future. Rule violation patterns documented herein demonstrate that there is room and urgent need for improvement in mitigating disease risks caused by tourists visiting the mountain gorilla of BINP. The present gorilla tourism model as implemented introduces substantial health risks for the very animals it tries to protect, and increasing tourist demand is likely to further increase risks unless substantial actions are taken to mitigate risks of disease transmission.

## Data Availability Statement

All datasets generated for this study are included in the article or available by request to the correspondent author.

## Ethics Statement

This study was conducted with permission from the Uganda Wildlife Authority and the Uganda National Council for Science and Technology in accordance with IRB and IACUC recommendations of the Ohio University Office of Research Compliance. All human subjects gave written informed consent in accordance with the Declaration of Helsinki. Protocol IRB#19.052 was approved by the Institutional Review Board Committee in the Ohio University Office of Research Compliance. Protocol IACUC# 19.049 was approved by the Institutional Animal Care and Use Committee in the Ohio University Office of Research Compliance.

## Author Contributions

NS, AW, and GK-Z contributed to conception and design of the study. AW carried out the field work. NS and AW analyzed the data. AW and NS assembled the first draft of the manuscript and figures.

### Conflict of Interest

The authors declare that the research was conducted in the absence of any commercial or financial relationships that could be construed as a potential conflict of interest.

## Footnotes

^1^Bwindi Impenetrable National Park General Management Plan, 2014-2023. www.ugandawildlife.org.
