# Supplementary material for: Lack of Rule-Adherence During Mountain Gorilla Tourism Encounters in Bwindi Impenetrable National Park, Uganda, Places Gorillas at Risk From Human Disease
Source: Front Public Health. 2020 Feb 13;8:1. doi: 10.3389/fpubh.2020.00001 (PMC7031198; doi:10.3389/fpubh.2020.00001)
Supplement: Supplementary file 1 [file Table_1.DOCX]

Supplementary table. Results of logistic regressions. Neither number of tourists per trekking group, nor duration of the trek to the gorilla-viewing location were significant in predicting the response variable (proportion of observations with 7 m rule violations out of the total observations in a given trek).

**Coefficient Estimate Std. Error t value Pr(>|t|)**

(Intercept) 0.646124 0.801002 0.807 0.4237

Trek duration -0.004019 0.002121 -1.895 0.0639

# of Tourists 0.101455 0.079396 1.278 0.2072
